# A Study on the Bacteriological Profile of Ascitic Fluids and Their Antibiotic Susceptibility Pattern in a Tertiary Care Hospital

**DOI:** 10.7759/cureus.49261

**Published:** 2023-11-22

**Authors:** Balaji Pachiyappan, Hemalatha S, Lidhiyah Sethuraman

**Affiliations:** 1 Microbiology, Government Medical College, Krishnagiri, Krishnagiri, IND; 2 Microbiology, Chengalpattu Medical College, Chengalpattu, IND; 3 Microbiology, Government Medical College, Nagapattinam, Nagapattinam, IND

**Keywords:** gram-negative bacilli, ascitic culture, ascitic fluid culture, bacterial infection, phenotypic methods, ascites

## Abstract

Background

Ascitic fluid culture remains an essential step in the management of all patients with ascites, regardless of their presenting complaints. Diagnostic paracentesis should not be delayed or prevent timely administration of antibiotics, particularly in unstable patients. Hence, it is an essential part of the surveillance system of every hospital to perform ascitic fluid culture and assess the antibiotic susceptibility patterns of bacterial isolates. In view of this perspective, the present study was conducted at Chengalpattu Medical College Hospital, Tamil Nadu, India.

Objective

The aim of the study is to determine the bacterial isolates of ascitic fluid samples and study their antibiotic susceptibility patterns.

Materials and methods

Ascitic fluids received in the central laboratory at the Department of Microbiology from various departments were included in this study. Preliminary identification of isolates was performed by direct Gram staining, acid-fast staining, and motility testing by the hanging drop method. Within one hour of receiving the samples, they were plated onto blood agar and MacConkey agar media and incubated for 18-24 hours at 37°C for isolation. Growth was checked, and species identification was done based on conventional methods. Antibiotic susceptibility testing was performed using the Kirby-Bauer disk diffusion method.

Results

In this study, a total of 100 ascitic fluid samples were collected, of which only eight (8%) showed growth. Among the eight isolates, six (75%) were Gram-negative bacilli (GNB). Four (66.66%) of the six GNB were *Klebsiella *spp., while the remaining two (33.33%) were *Escherichia coli*. Both Gram-positive cocci were *Staphylococcus aureus*. All the GNB isolates were susceptible to meropenem, piperacillin-tazobactam, and ceftriaxone, with varying susceptibilities to other drugs. Both Gram-positive isolates were found to be methicillin-sensitive *Staphylococcus aureus.*

Conclusion

GNB were the predominant organisms in cases of ascitic fluid infection, and they showed 100% susceptibility to carbapenem drugs (especially meropenem), piperacillin-tazobactam, and ceftriaxone. All these drugs can be kept in reserve for serious infections. Amikacin and gentamicin showed promising susceptibility. These drugs can be started empirically with patients on admission before performing culture. Drug adjustments may be later made based on culture reports.

## Introduction

Ascites is a clinical manifestation of an underlying pathological process that results in the accumulation of fluid within the peritoneal cavity, typically exceeding 25 mL. It is a significant and often debilitating complication seen in various medical conditions, and its pathophysiology is multifaceted and complex. Its mortality rate ranges from 15% in one year to 44% in five years [1-3].

Overwhelmingly, the most common cause of ascites is portal hypertension related to cirrhosis. Several primary disorders of the peritoneum and visceral organs can also cause ascites. Other causes include malignancy, cardiac failure, tuberculosis, and pancreatic diseases. It can also develop following paracentesis [4].

Infectious causes of ascites can be present in several patients, which should be differentiated from other causes. Ascitic fluid infection over existing comorbidities usually impairs the prognosis of the patient. Gram‐negative bacilli (GNB) constitute the major cause of ascites (about 60%-75%), of which *Escherichia coli* is the commonest. Gram‐positive cocci (GPC) have generally accounted for less than 25% of ascites cases [5,6].

However, in recent times, GPC such as *Staphylococcus aureus* and *Enterococcus *spp. are on the rise. This change has had a direct impact on the diagnosis, treatment, and outcomes of patients [7]. Extended-spectrum β‐lactamase‐producing GNB (*E. coli and Klebsiella*) (73%), followed by methicillin‐resistant *S. aureus* and vancomycin‐resistant *Enterococcus*, is the most common multidrug-resistant bacterial isolate found in ascitic fluid infection.

When patients present with ascites, it is recommended that diagnostic paracentesis be performed to characterize the fluid. This should include the determination of total protein and albumin content, blood cell counts, and cultures. Hence, ascitic fluid culture remains an essential step in the management of all patients with ascites, regardless of their presenting complaints. Diagnostic paracentesis should not be delayed or prevent timely administration of antibiotics, particularly in unstable patients. Hence, it is an essential aspect of the surveillance system of every hospital to perform ascitic fluid culture and assess the antibiotic susceptibility patterns of bacterial isolates in ascitic cases.

## Materials and methods

This is a hospital-based cross-sectional study conducted over a year, from October 2022 to September 2023.

Inclusion and exclusion criteria

All adult patients clinically diagnosed with ascites, irrespective of sex and other comorbid conditions, were included. Pediatric patients and consecutive samples of the same patients were excluded from the study. Informed consent was obtained from the participants included in the study. The Institutional Ethical Committee at Chengalpattu Medical College issued approval for the study before its commencement (approval number CHMC/IEC/191/2022).

Sample collection

Ascitic fluid samples from 100 patients fulfilling the inclusion criteria were included in this study. Following universal precautions and the wearing of appropriate personal protective equipment, samples were collected under strict aseptic precautions. Prelabeled sterile collection tubes were used. Patients were placed in a supine position at the edge of a bed, with the trunk elevated to 45°. The site (midline 3-4 cm below the umbilicus and halfway between the pubic symphysis and the umbilicus) for sample collection was marked prior to collection, followed by skin disinfection using 70% isopropyl alcohol and povidone-iodine. A volume of 50 mL of ascitic fluid was collected and divided into three parts for other analyses. One part was transported to the microbiology laboratory immediately.

Macroscopic and microscopic examination

The macroscopic appearance of the specimen was observed for the color of the effusion and the presence of blood. Samples received were subjected to preliminary identification by direct Gram staining, acid-fast staining, and motility testing by the hanging drop method.

Culture

After preliminary identification and within one hour of receiving the samples, they were plated onto blood agar and MacConkey agar media and incubated for 18-24 hours at 37°C for isolation. Growth was checked after overnight incubation, and species identification was done by standard biochemical techniques.

Antimicrobial susceptibility testing

The isolated organisms were subjected to antimicrobial susceptibility testing by disk diffusion using the modified Kirby-Bauer technique. Three to four morphologically similar colonies were suspended in peptone water and incubated at 37°C for two hours. The turbidity of the test suspension was standardized to 0.5 McFarland units. The suspension was inoculated on a Mueller-Hinton agar plate with a sterile cotton wool swab by the lawn culture method. After brief drying, antibiotic disks (about six disks per 100 mm plate) were placed with sterile precautions. Then, the plate was incubated at 37°C for 24 hours and interpreted the next day as per the Clinical and Laboratory Standards Institute guidelines. All the above tests were performed in conjunction with positive, negative, and test controls as necessary according to the standard guidelines.

The panel of drugs used for antibiotic susceptibility testing for GPC were penicillin 10 U, erythromycin 15 µg, clindamycin 2 µg, cotrimoxazole 1.25/23.75 µg, tetracycline 30 µg, cefoxitin 30 µg, linezolid 30 µg, and vancomycin.

For GNB, the following were used: ampicillin 10 µg, gentamicin 10 µg, amikacin 30 µg, ceftriaxone 30 µg, cotrimoxazole 1.25/23.75 µg, ciprofloxacin 5 µg, piperacillin-tazobactam 100/10 µg, and meropenem 10 µg.

Statistical analysis

We recorded the study data in Microsoft Excel 2019 (Microsoft Corporation, Redmond, WA). The frequencies and percentages of the organisms isolated were tabulated.

## Results

In this study, a total of 100 ascitic fluid samples were collected from various departments at Chengalpattu Medical College. The study included both genders (62 males and 38 females) in the age group of 18 years and above. Table 1 presents the age distribution of the patients.

**Table 1:** Age distribution of patients with ascites

Age distribution	Number of patients with ascites	Percentage (%)
Up to 20 years	3	3
21-30 years	9	9
31-40 years	22	22
41-50 years	28	28
51-60 years	21	21
61-70 years	14	14
71-80 years	1	1
81-90 years	2	2
Total (n=100)	100	100

Of the 100 ascitic fluid samples collected, eight (8%) produced a positive bacterial culture, and 92 (92%) produced a negative bacterial culture (Table 2).

**Table 2:** Percentages of positive and negative growth of ascites

Ascites	Growth positive	Growth negative
Total (n=100)	8 (8%)	92 (92%)

**Figure 1:**
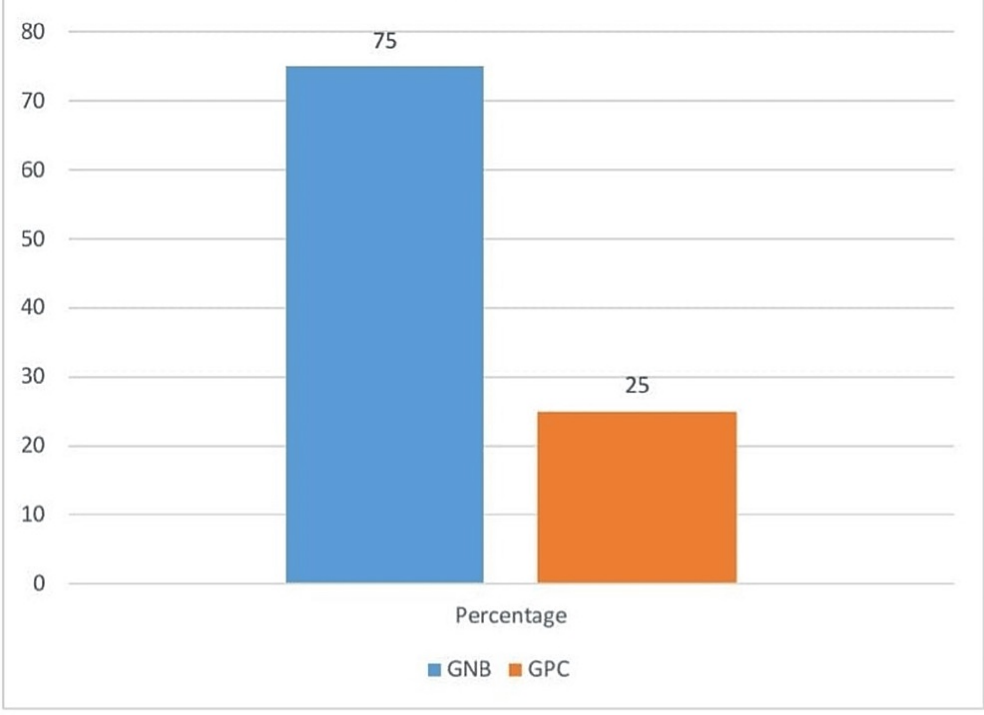
Distribution of pathogens GNB: Gram-negative bacilli, GPC: Gram-positive cocci

For the samples of the 62 male patients with ascites, six (9.67%) showed positive growth, while 56 (90.32%) did not. For the samples of the 38 female patients with ascites, two (5.26%) showed positive growth, while 36 (94.73%) did not. Out of the eight isolates, six (75%) were GNB, the most common organism group in infection cases in the patients (Figure 1).

**Figure 2:**
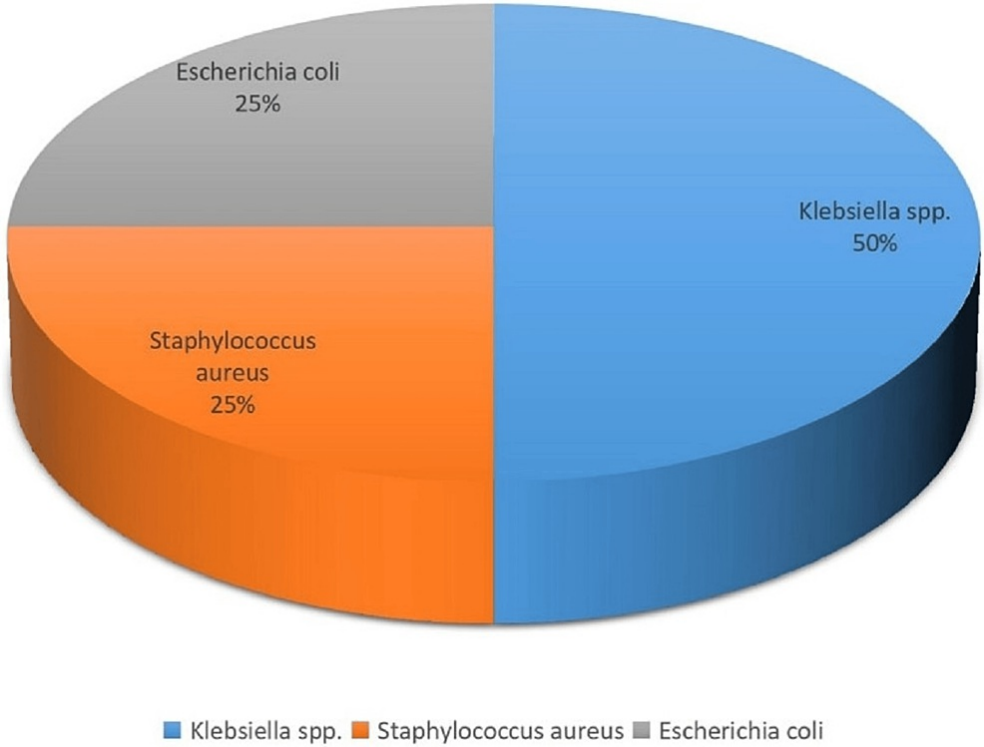
Species distribution of pathogen in ascitic fluid samples

Regarding the GNB, four (66.66%) were *Klebsiella *spp., while the other two (33.33%) were *E. coli*. Among the four *Klebsiella *spp., three were *Klebsiella pneumoniae*, and the last one was *Klebsiella oxytoca. *The remaining two positive growth isolates were *S. aureus*, a GPC (Figure 2).

**Figure 3:**
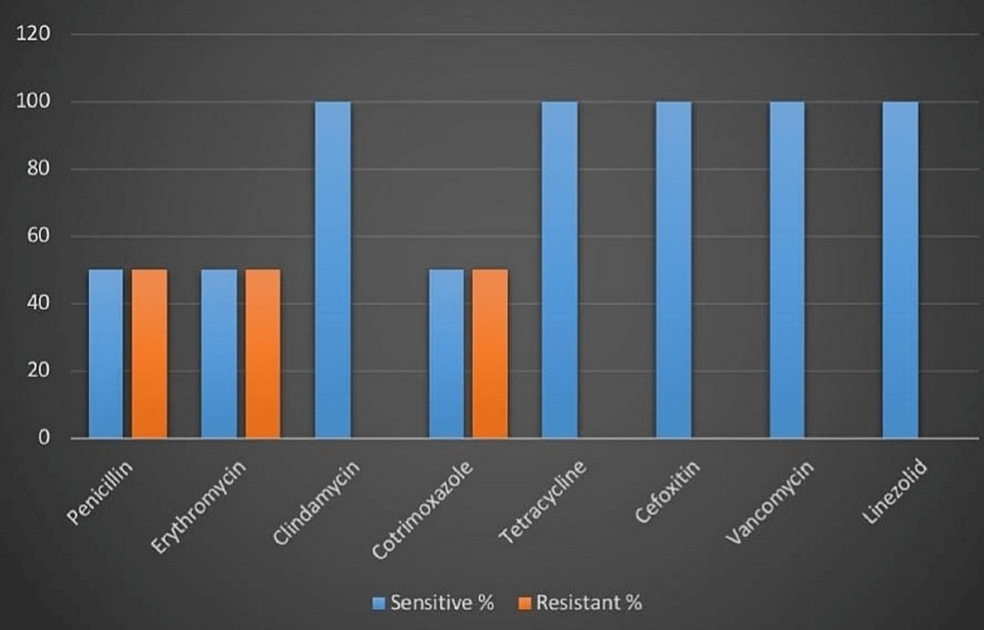
Antibiotic susceptibility pattern of Staphylococcus aureus (n=2)

In this study, both *S. aureus* isolates were susceptible to clindamycin, tetracycline, vancomycin, and linezolid, while only one isolate was susceptible to penicillin and erythromycin. Both isolates were methicillin-sensitive* Staphylococcus aureus* since none of them showed resistance to cefoxitin (Figure 3).

**Figure 4:**
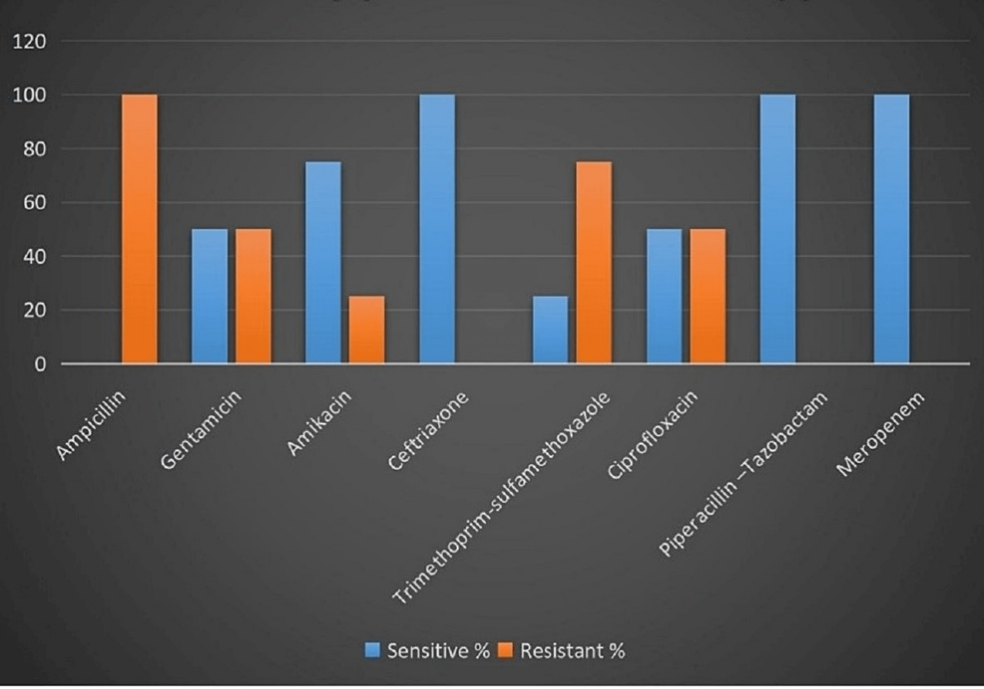
Antibiotic susceptibility pattern of Klebsiella spp. (n=4)

*Klebsiella *spp. showed higher sensitivity for ceftriaxone (n=4, 100%), piperacillin-tazobactam (n=4, 100%), and meropenem (n=4, 100%), followed by amikacin (n=3, 75%), ciprofloxacin (n=2, 50%), gentamicin (n=2, 50%), and trimethoprim-sulfamethoxazole (n=1, 25%). All four isolates showed resistance to ampicillin, which may be attributed to the intrinsic resistance of *Klebsiella *spp. to the drug (Figure 4).

**Figure 5:**
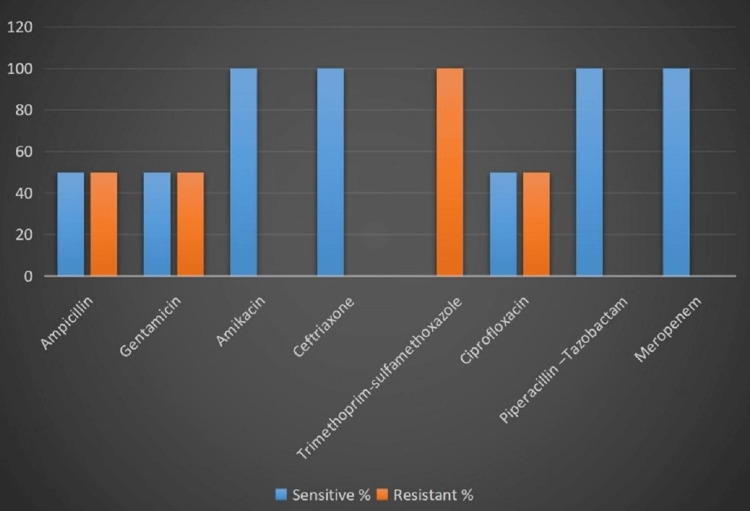
Antibiotic susceptibility pattern of Escherichia coli (n=2)

The *E. coli* isolates showed higher sensitivity for ceftriaxone (n=2, 100%), amikacin (n=2, 100%), piperacillin-tazobactam (n=2, 100%), and meropenem (n=2, 100%), followed by ciprofloxacin (n=2, 50%), gentamicin (n=2, 50%), and ampicillin (n=2, 50%). Both isolates were resistant to trimethoprim-sulfamethoxazole (Figure 5).

## Discussion

Ascites, with its diverse clinical syndromes, remains one of the most common but widely misunderstood and challenging body fluid infections encountered in clinical practice. The risk of developing ascites increases significantly with age, even when other risk factors are present. In patients with cirrhosis and ascites that develop severe enough for hospitalization, even spontaneous bacterial peritonitis can occur in up to 30% of individuals and can have a 25% in-hospital mortality rate due to the emergence of multidrug-resistant organisms [8].

Bacterial translocation is the presumed mechanism for the development of infection in ascites, with gut flora traversing the intestine into mesenteric lymph nodes, leading to bacteremia and seeding of the ascitic fluid. The most common organisms are *E. coli* [9] and other gut bacteria; however, Gram-positive bacteria, including *Streptococcus viridans*, *S. aureus*,and *Enterococcus* spp., can also be found.

If more than two organisms are identified, secondary bacterial peritonitis due to a perforated viscus should be considered. Bedside cultures should be obtained when ascitic fluid is tapped. Patients with ascites may present with fever, altered mental status, elevated white blood cell count, and abdominal pain or discomfort, or they may present without any of these features. Therefore, it is necessary to have a high degree of clinical suspicion, and peritoneal taps are important for making the diagnosis [10,11].

A total of 100 patients, those who fulfilled the inclusion criteria, were included in this study. Out of the 100 patients, 62 were male and 38 were female, and most (n=28, 28%) belonged to the age group of 41-50 years, followed by 31-40 years (22%). Among the 100 patients with ascites, three (3%) were in the age group of up to 20 years, nine (9%) were in the age group of 21-30 years, 22 (22%) were in the age group of 31-40 years, 28 (28%) were in the age group of 41-50 years, 21 (21%) were in the age group of 51-60 years, 14 (14%) were in the age group of 61-70 years, one (1%) was in the age group of 71-80 years, and two (2%) were in the age group of 81-90 years.

Of the 100 samples collected, eight showed positive growth in culture. Among the eight isolates, six (75%) were GNB, and two (25%) were GPC [5-8]. Among the 62 male patients with ascites, only six (9.67%) showed positive growth in culture, and among the 38 female patients with ascites, only two (5.26%) showed positive growth. Among a total of eight positive patients, six (75%) were male, and two (25%) were female. Among the 92 negative patients, 56 (60.86%) were male, and 36 (39.13%) were female.

Among the eight culture-positive cases, the most common organism isolated was *Klebsiella *spp. in four cases, followed by *E. coli *in two cases and *S. aureus* in two cases, which is in contrast with studies of Purohit et al. [9] and Kirplani et al. [5], where *E. coli* was the commonest among GNB. A study conducted by Bhat et al. [12] shows *Klebsiella *spp. was the commonest organism, which is consistent with this study.

In relation to the resistance pattern, all six GNB isolates were susceptible (100%) to meropenem, piperacillin-tazobactam, and ceftriaxone. This is in contradiction with the studies by Kirplani et al. [5], Dever et al. [8], and Li et al. [7], where second- and third-generation cephalosporins showed the highest resistance to GNB isolates [12-14].

This study did not detect any extended-spectrum or AmpC β‐lactamase‐producing pathogens among the GNB isolates, and in the same way, no methicillin‐resistant *S. aureus* was detected. This finding is in contravention of the study by Dever et al. [8], which noted that multidrug-resistant organisms have become common pathogens in cases of spontaneous bacterial peritonitis.

Diagnostic paracentesis should always be performed for all cases of ascites, irrespective of cause [15]. A microbiological study by means of ascitic fluid culture and antibiotic susceptibility testing should also be performed to find out the causative organism and its sensitivity pattern in cases of infected ascites and thereby improve the prognosis of patients.

In our study, the Gram-negative bacterial isolates were 100% sensitive to meropenem, piperacillin-tazobactam, and ceftriaxone, and better sensitivity was observed to amikacin and gentamicin. Multidrug-resistant organisms were not isolated in this study. An extended version of this study with a larger sample size and a longer duration can be done in the future for a better perspective on infected cases of ascites.

## Conclusions

Gram-negative organisms were the predominant organisms in cases of ascitic fluid infection, and they showed 100% susceptibility to meropenem, followed by piperacillin-tazobactam and ceftriaxone. All these drugs can be kept in reserve for serious infections. Amikacin and gentamicin also showed better sensitivity and were cost-effective. These drugs can be started empirically with patients on admission and can be modified later as per culture sensitivity reports.

Bacterial ascites have become a major complication in patients with liver diseases. Infected peritoneal dialysate appears to be quite comparable to spontaneous bacterial peritonitis. Both forms of peritonitis involve small numbers of bacteria. Cultures of large volumes of fluid are required to detect the offending organism. Routine culture methods of ascitic fluid detect bacterial agents in less than 10% of the population. The cause for this low level of detection can be attributed to the small number of microorganisms present in it. As an alternative to conventional culture methods, bedside inoculation of ascitic fluid in blood culture bottles that contain brain heart infusion broth in sufficient volumes can act as an enrichment medium and provide a better nutritious environment for the bacterial agents causing infection than the ascitic fluid itself. This may result in a higher detection rate of microorganisms in most cases of infected ascites.
